# Variability in Seroprevalence of Rabies Virus Neutralizing Antibodies and Associated Factors in a Colorado Population of Big Brown Bats (*Eptesicus fuscus*)

**DOI:** 10.1371/journal.pone.0086261

**Published:** 2014-01-22

**Authors:** Thomas J. O’Shea, Richard A. Bowen, Thomas R. Stanley, Vidya Shankar, Charles E. Rupprecht

**Affiliations:** 1 United States Geological Survey, Fort Collins Science Center, Fort Collins, Colorado, United States of America; 2 Department of Biomedical Sciences, Colorado State University, Fort Collins, Colorado, United States of America; 3 Centers for Disease Control and Prevention, Atlanta, Georgia, United States of America; 4 Ross University School of Veterinary Medicine, Basseterre, Saint Kitts, West Indies; 5 The Global Alliance for Rabies Control, Manhattan, Kansas, United States of America; CSIRO, Australia

## Abstract

In 2001–2005 we sampled permanently marked big brown bats (*Eptesicus fuscus*) at summer roosts in buildings at Fort Collins, Colorado, for rabies virus neutralizing antibodies (RVNA). Seroprevalence was higher in adult females (17.9%, n = 2,332) than males (9.4%, n = 128; *P* = 0.007) or volant juveniles (10.2%, n = 738; *P*<0.0001). Seroprevalence was lowest in a drought year with local insecticide use and highest in the year with normal conditions, suggesting that environmental stress may suppress RVNA production in big brown bats. Seroprevalence also increased with age of bat, and varied from 6.2 to 26.7% among adult females at five roosts sampled each year for five years. Seroprevalence of adult females at 17 other roosts sampled for 1 to 4 years ranged from 0.0 to 47.1%. Using logistic regression, the only ranking model in our candidate set of explanatory variables for serological status at first sampling included year, day of season, and a year by day of season interaction that varied with relative drought conditions. The presence or absence of antibodies in individual bats showed temporal variability. Year alone provided the best model to explain the likelihood of adult female bats showing a transition to seronegative from a previously seropositive state. Day of the season was the only competitive model to explain the likelihood of a transition from seronegative to seropositive, which increased as the season progressed. We found no rabies viral RNA in oropharyngeal secretions of 261 seropositive bats or in organs of 13 euthanized seropositive bats. Survival of seropositive and seronegative bats did not differ. The presence of RVNA in serum of bats should not be interpreted as evidence for ongoing rabies infection.

## Introduction

The presence of rabies virus neutralizing antibodies (RVNA) in serum of insectivorous bats of North America has been documented for over 50 years (*e.g.* [1]–[4]). However, initial investigations could not determine to what degree the presence of serum RVNA signaled past exposure, immunity, abortive infection, subclinical, or incubation phases of rabies [1], [3], [5]. Additionally, past serological surveys for RVNA in North American insectivorous bats were cross-sectional, in that wild bat populations were sampled once (sometimes terminally) and not marked for subsequent sampling. Historically, such serological surveys also concentrated on samples from small numbers of bat colonies for which there was limited ecological background information. More recent serological studies in Europe have indicated the presence of serum antibodies to other bat lyssaviruses in several species of insectivorous bats, usually at low prevalence (reviewed by Schatz et al. [6]). These latter studies included cross-sectional sampling at multiple locations and colonies [7]–[11], limited longitudinal sampling of marked individual bats [9], [12], [13] and analysis of ecological factors associated with seroprevalence [10], [13].

Herein we report on both cross-sectional and longitudinal prevalence of RVNA in serum samples of big brown bats (*Eptesicus fuscus*) roosting commensally with humans in the urbanizing setting of Fort Collins, Colorado, U.S.A. Big brown bats are the most common species of bat submitted for rabies diagnostic testing in passive public health surveillance programs in the U.S. and in Colorado [14]–[16]. Recent complementary studies of rabies pathogenesis in captive big brown bats have included measurements and interpretations of the presence of RVNA based on laboratory experiments [17]–[22]. The big brown bat population we sampled roosts commensally with people in buildings and has been characterized by a number of concurrent ecological [23]–[25], demographic [26], [27], and genetic studies [28]. This surveillance, laboratory, and field background provides additional information of potential importance for understanding the significance of serum RVNA in big brown bats (see *Methods* for more detail).

Our study focuses on testing the hypothesis that the presence of RVNA in serum of bats is indicative of past exposure of bats to rabies and is not evidence for an ongoing rabies infection per se. Our first objective in the present paper is to provide an in-depth cross-sectional profile of RVNA seroprevalence in a big brown bat population, and to test variation in RVNA seroprevalence based on sex, age, year of study, and roosting colony. Secondly, we describe longitudinal variability in the presence of RVNA in individually marked bats, and test multiple competing hypotheses about the relative importance of a number of biological and environmental factors that have potential influence on the serological status of individual bats over time. A cross-sectional study of Mexican free-tailed bats (*Tadarida brasiliensis mexicana*) roosting in large colonies in caves and bridges in Texas has reported interactions among ecological factors and variance in seroprevalence during a single year, with important associated factors including roost and season [29]. Recent studies of other diseases in unrelated species of wildlife have suggested that temporal changes in the immune status of individuals can occur in relation to a variety of environmental stress-related factors (*e.g.* [30], [31]). Our longitudinal analyses attempt to explore the importance of such influences on RVNA seroprevalence in big brown bats at the Colorado study area. Our final objective was to shed additional light on the interpretation of serum RVNA in bats and on our initial hypothesis by sampling seropositive bats for evidence of rabies virus (RV) RNA in oral secretions and tissues, and to describe the survival of seropositive bats over time.

## Methods

### Study Area and Supporting Background Research on the Bat Population

We studied big brown bats at Fort Collins, Colorado, during summers 2001–2005. Many aspects of the big brown bat population at Fort Collins were studied intensively simultaneous to our serological sampling, and a more detailed description of the study area appears in [25]. Big brown bats are the most common bat in Fort Collins, as in many urban areas in North America (reviewed in [25]). The population of big brown bats at Fort Collins roosts only in buildings, and the bats migrate to higher elevations in the adjacent Rocky Mountains for winter hibernation in rock crevices [23], [25]. Summer roost locations occurred throughout the city, and were previously mapped and characterized [24], [25]. Big brown bats generally show high fidelity to roosts, including high natal philopatry [26], but will move to neighboring roosts during periods of hot weather [32]. Numbers of adults counted emerging from roosts ranged from 10 to 219, with a geometric mean of 47 bats (95% Confidence Interval [*CI*] 39–56; [25]). Bats from multiple colonies concentrated foraging at overlapping riparian areas, and extensive dietary heterogeneity was inferred based on isotopic analysis of hair samples of many individuals from several roosts [25] [33]. Two mitochondrial DNA haplotypes of big brown bats are found at the study area but interbreed and mingle within colonies [28]. Demographic characteristics of the population include: a litter size of 1 with occasional twinning, a survival rate of 0.67 from volancy to the second summer, and breeding probabilities of 0.64 for one-year-old females and 0.95 for older females [26]. Annual adult survival averaged 0.79 with the best candidate models for explaining survival including roost and year; winter survival was higher than survival in summer, and population growth rates were positive [27].

The RVs in Colorado big brown bats were genotyped and include primarily two clades based on nucleoprotein (N) gene sequences [34]. Public health surveillance records for rabies in big brown bats in the region were analyzed and include the regular occurrence of rabid bats and exposure of humans and domestic animals to rabid bats [14], [25]. About 2.5% of 199 big brown bats taken from the population eventually developed rabies when held captive for six months [21]. Big brown bats captured at the study area were experimentally subjected to aerosol exposures to one of the RV variants circulating in their wild population and showed serum antibody responses, but succumbed to subsequent intramuscular inoculation with a different variant [20]. In a separate experiment with captive bats from the study population, most (78%) bats inoculated with varying dosages of the same variant responded by producing serum RVNA but none developed rabies [22]. Eight of 11 bats that were seronegative at capture developed rabies when inoculated with a second variant found in the population, but none of the bats that were seropositive at the time of capture developed rabies when inoculated with this second variant [22]. The survival of experimentally inoculated seropositive bats in these studies suggests some degree of protection is afforded by RVNAs. The dynamics of RV infection in this population were modeled mathematically by George et al. [35], who found that seasonally high adult survival in winter favors maintenance of the host population, while the virus is maintained by long incubation periods and overwinter dormancy followed by a seasonal birth pulse of immunologically susceptible young.

**Figure 1:**
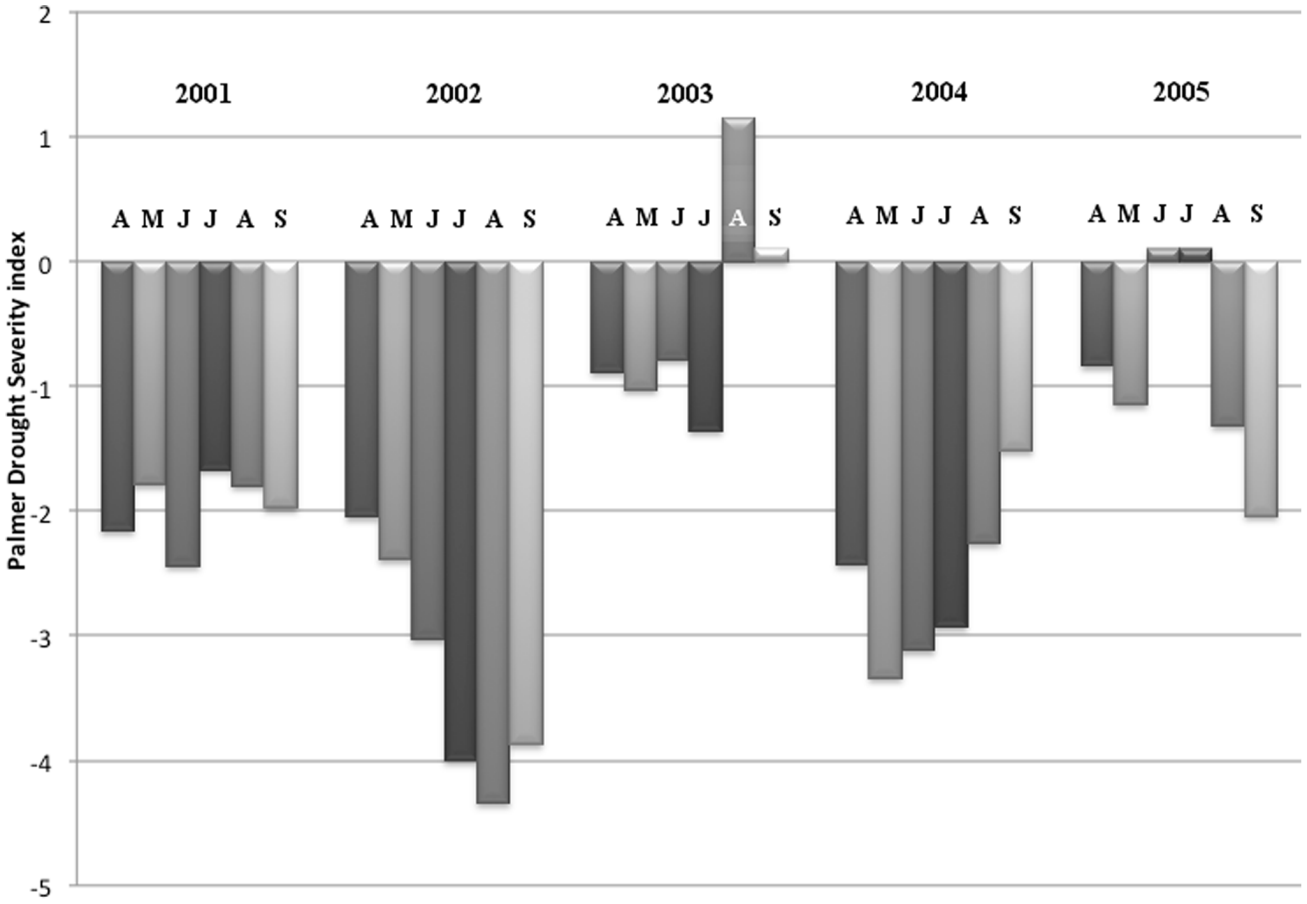
Palmer Drought Severity Index for the spring and summer months in the region including Fort Collins, Colorado, 2001–2005. Relative monthly drought is indicated by the depth of bars below zero.

Our study was conducted during a long drought that varied in intensity by year (Figure 1), as measured by the Palmer Drought Severity Index (PDSI) for the warm-season period of bat activity (April-September) for Colorado Zone 11, north Front Range, and adjacent plains [36]. The PDSI is a standardized method for measuring intensity, duration, and spatial extent of drought based on precipitation, air temperature, and local soil moisture, with values ranging from −6.0 (extreme drought) to +6.0 [37]. Insect prey and demography of bats can be depressed during droughts [38]–[40], making year of sampling an important environmental variable. In addition to the PDSI, we also compiled and summarized daily temperature data for the summer sampling periods in each year of study using data available for Fort Collins [41].

### Capture, Marking, and Sampling of Bats

We captured bats in mist nets over water at night, applied radio transmitters before releasing, and located roosting colonies by searching for radio signals while driving city streets during the day [25], [42]. We captured bats haphazardly at roost entrances as they emerged after dusk using mist nets, harp traps, funnel traps, and hand-held nets (we followed guidelines of Sheffield et al. [43] and usually avoided entering maternity roosts to capture bats). We sampled bats at five roosts for each of the five summers, and sampled additional bats at 17 other roosts for 1–4 summers (bats were often excluded from roosts by building owners, preventing re-sampling every summer; see [25] for details). We repeated captures of bats at the same roosts 1–8 times each summer with a mean interval of 20.5 days (95% confidence interval [*CI*] 20.0–23.0 days) [25] but could not always recapture the same individuals. Bats were transported to the laboratory in individual cloth bags nested within disposable cups with lids. At the laboratory each bat was examined, measured, and permanently marked with passive integrated transponders (PIT tags) [44]. Volant juveniles were distinguished from adults based on lack of ossification of phalangeal epiphyses [45]. This allowed assigning of accurate ages to bats first marked as volant juveniles when they were captured in subsequent years. Entry points of selected roosts were continuously monitored with PIT tag readers to record presence of individuals for survival analyses [42], [44], [46].

Colony sizes were estimated through emergence counts [47] at dusk. We provide these data for each roost to indicate relative size of colonies and base them on the highest counts of any year during the month of June, when colonies had formed but most juveniles were not volant (based on captures at roosts). The counts thus are assumed to represent adults only. Evening emergence counts can show daily variability because big brown bat colonies may occupy a number of alternate neighboring roosts (*e.g*. [32], [48]. We did not make numerous replicate counts at roosts to quantify this variability. Instead we used evening emergence counts only to document the approximate magnitude of colony sizes.

Captured bats were selected for serological sampling randomly in 2001–2003; beginning in 2003 we gave priority to sampling bats that had been sampled previously, with additional bats sampled at random as time allowed. Blood was obtained through interfemoral vessels as described by Wimsatt et al. [44], except that anesthesia was discontinued because there was no difference in survival between anesthetized, unanaesthetized, and unbled bats [49]. Oropharyngeal secretions of bats were sampled at the laboratory using cotton-tipped swabs inserted into 0.5 ml of BA-1 medium (Minimal Essential Medium with Hanks’ balanced salt solution containing 0.05 M Tris buffer at pH 7.6, 1% Bovine Serum Albumin, 0.35 g/L of Sodium bicarbonate, 50 mg/L Gentamicin, and 2.5 mg/L Amphotericin B). Serum and oropharyngeal swabs were frozen at −80 C until shipment to the Centers for Disease Control and Prevention (CDC), Atlanta, Georgia for testing [17], [50]. Capture, marking, sampling, and euthanasia of bats were approved by the Institutional Animal Care and Use Committees of Colorado State University and the United States Geological Survey. Bats were captured under authority of a scientific collecting license issued by the Colorado Division of Wildlife.

### Laboratory Analyses

Serum samples were analyzed at the Rabies Laboratory, CDC, Atlanta, Georgia for determination of RVNA using the Rapid Fluorescent Focus Inhibition Test (RFFIT), a standard RV neutralization assay used in diagnostic laboratories [17], [51]. RV neutralization activity in big brown bat serum is associated with the immunoglobulin G antibody fraction rather than with non-specific serum components [17]. Rabies Immune Globulin International Standard from the World Health Organization was used as positive control and a pool of unvaccinated mouse sera served as negative control in all assays. The RFFIT results were expressed as endpoint titers. Titers <5 were considered negative, and samples with RFFIT titers >5 were considered positive. Negative samples were excluded prior to statistical summarization and analysis if serum volumes were less than 12 μl due to concerns about assay sensitivity.

An aliquot (100 µL) of each oropharyngeal swab sample was tested for the presence of RV RNA using a nested RT-PCR assay targeting the N gene [17]. Samples from brain and salivary glands of seropositive bats were also assayed from euthanized bats with this technique. Brain tissue of suspected rabid bats was assayed for RV antigen using the Direct Fluorescent-Antibody (DFA) test. Bat brain impressions from frozen tissue were processed and examined for RV antigen by using a fluorescein isothiocyanate-labeled anti-rabies antibody [52], [53].

### Data Analysis

We provide simple summary statistics to describe seroprevalence patterns in various groupings of bats: proportion seropositive, number of bats sampled, and 95% *CI* for proportions with correction for continuity [54]. Seroprevalence at other roosts that were sampled only in 2003 are reported elsewhere [50]. We use study numbers for roosts listed in summary statistics, but to protect confidentiality of building owners and occupants we do not provide details of roost locations or names. For cross-sectional summaries by year, we included only one serological value in our computations for an individual sampled more than once in that summer. Because we were interested in numbers of individual bats that showed RVNA (were seropositive), and if serial assays of an individual included both positive and negative values within a summer we included only the positive value in the cross-sectional summaries. Similarly, because we were interested in past evidence of exposure to RV, in calculating seroprevalence in known-age adult bats we present data as cumulative over time (proportion of individuals of known age with a history that included a seropositive sample). In some cases we were interested in whether the proportion of seropositive individuals differed across groupings (*e.g*., age classes, years). To evaluate this we used the Marascuilo procedure [55] to compute *χ* ^2^ statistics for pair-wise comparisons among proportions.

Longitudinal statistical samples involved many individuals. For illustrative purposes, we provide results of individual longitudinal serological sampling for 20 adult female bats sampled 5 or more times to show examples of temporal variability in expression of RVNA. We chose all individuals with 6 or more sampling dates (n = 7), and randomly selected 13 additional bats from among 37 individuals bled 5 times each over the course of the study. We also analyzed serological data from adult female bats using logistic regression in a generalized linear models context [56] under three distinct frameworks: 1) we modeled the serological status of the bat only on the date it was first sampled (*y* = 1 if seropositive, *y* = 0 otherwise); 2) we conditioned on bats that were seronegative on a capture date, then modeled the status of the bat on the next occasion it was sampled (*y* = 1 if it transitioned to seropositive, *y* = 0 otherwise); and 3) we conditioned on bats that were seropositive on a capture date, then modeled the status of the bat on the next occasion it was sampled (*y* = 1 if it transitioned to seronegative, *y* = 0 otherwise). Under frameworks 2 and 3 we only used data from bats that had a prior history of exhibiting RVNA as adults to ensure that we had evidence they could be capable of mounting a RVNA response. For all three sets of analyses we used a logit link to incorporate covariates for use as predictor variables, and evaluated an *a priori* set of candidate models that we believed could potentially explain variation in serological status. The variables in our candidate set of models under all three frameworks were: year of sampling; number of fresh open puncture holes in the wing that were the approximate size of big brown bat canine tooth tips (assumed to be a measure of recent exposure to bites from another bat, but not definitively known); and mean minimum and maximum ambient temperatures for the 5 days preceding sampling. None of the explanatory variables appearing in a particular candidate model had a correlation coefficient with an absolute value greater than 0.29. Big brown bats are heterothermic (body temperature of inactive bats can vary with environmental temperature); body temperature and metabolism of heterothermic mammals can affect immune function and expression of antibodies [57]–[60]. We chose thermal regimes for the 5 days prior to sampling considering that longer intervals might mask the importance of recent mean maxima or minima, and because the half-lives of immunoglobulin antibodies in experimental mice are short, ranging from 0.4 days to 11.7 days (*e.g*. [61], [62]). Two additional variables used in the candidate set of models were day of season (beginning 6 April each year, approximating when bat activity begins at the study area [25], [35]); and reproductive status (pregnant, lactating, post-lactating or non-reproductive; [26]). Under frameworks 2 and 3, we added a variable representing the number of days elapsed between the two sampling occasions. Our candidate sets of models included those representing potential additive and interactive effects of pairs of variables. We did not include a roost variable and its interactions in our final set of candidate models, based on the low ranking of roost in preliminary exploratory analyses. We limited these analyses to the years 2002–2005 because sampling in the first year of study (2001) was biased towards dates later in the season.

We compared alternative models within the three candidate sets of models using Akaike’s Information Criterion corrected for small sample size (AIC*_c_*), and computed model weights (*w*) [63]. The ΔAIC*_c_*, computed as the difference between the AIC*_c_* value of the model of interest and the AIC*_c_* of the best model (*i.e*., AIC*_c_*_min_), along with model weights (*w*), provided a measure of the relative strength of evidence for each model given the data and our candidate set of models. Models with ΔAIC*_c_* ≤2 were considered to have substantial support, with the exception of those that differed from the best model by one additional parameter and which had essentially the same values of the maximized log-likelihood as the best model [63], [64]. Because these latter models provide no net reduction in AIC*_c_* [64], we excluded them from consideration. Herein we report only results for models with ΔAIC*_c_* ≤2.

We estimated apparent annual survival (*φ*) over 2001–2005 for female bats that were seropositive as adults at any time in 2001–2003 in comparison with adult females that were only seronegative when sampled in 2001–2003. We based the survival estimates on encounters of tagged bats with PIT tag readers in fixed positions at entrances of five roosts monitored from 2001–2005 [46]. Survival was calculated using Program Mark with differences between the seropositive and negative groups determined by *χ* ^2^ goodness-of-fit tests in Program Release Test 1 [49], [65]. A more detailed multi-model analysis of factors influencing adult survival that does not include serological status was previously published [27]. We also summarize numbers of seropositive bats known alive from encounters by tag readers or captures after lengthy intervals during this study, and in nets during an unrelated subsequent study [33] of the same population.

## Results

### Seroprevalence in Volant Juveniles and Known-Age Adults

We sampled 738 marked volant juveniles (including 74 sampled twice as juveniles at variable intervals): 75 were seropositive (10.2%; Table 1). The proportion seropositive was similar between volant juvenile females and volant juvenile males (Table 1). We sampled 388 volant juvenile females 434 times: 39 were seropositive (10.1%; Table 1). Serial sampling involved two samples each from 46 tagged volant juvenile females: 38 juvenile females were negative on both sample dates (25.8±9.5 days apart), two were positive on both dates (11 and 27 days apart), five were first positive, then negative on second sampling dates (34.4±8.4 days apart), and one was first negative, then positive on a second sampling date (32 days apart). We sampled 350 marked volant juvenile males 378 times: 36 were seropositive (10.3%; Table 1). Serial sampling involved two samples each from 28 volant juvenile males during a single year. Twenty-four were negative on both samples (17.3±7.2 days apart), 3 were first positive, then negative on second sampling dates (17, 22, and 34 days elapsed), and one was first negative, then positive (10 days elapsed).

**Table 1:** Seroprevalence of rabies virus neutralizing antibodies in known-age, marked individual big brown bats (*Eptesicus fuscus*), Fort Collins, Colorado, 2001–2005.

VJF	VJM	VJ	1 yr-old F	2 yr-old F	3 yr-old F	4 yr-old F	≥5 yr-old F
10.1%	10.3%	10.2%	14.3%^a^	27.1%^ab^	20.8%^ab^	47.8^b^	24.5^ab^
7.3–13.6	7.4–14.1	8.1–12.6	9.2–21.5	18.8–37.3	11.3–34.5	27.4–68.9	16.6–34.4
388	350	738	140	96	53	23	98

Proportions with superscripts in common for adult (≥1 year old) bats are not significantly different (*P*>0.05). Values are seroprevalence (% seropositive), 95% confidence interval for seroprevalence, and sample size (number of unique individuals sampled). Abbreviations: F =  female, M = male, VJ = volant juvenile.

We sampled 330 known-age (first tagged as juveniles) adult bats of both sexes 503 times. Only 19 were males, age 1 (n = 14) or 2 (n = 5) years old, none of which was seropositive. Adult females (n = 410) showed differences in cumulative seroprevalence with age (Table 1, *χ* ^2^ = 15.4, 4 d.f., *P* = 0.004) due primarily to the contrast between 1-year-old and 4-year-old bats (Table 1; *χ* ^2^ = 9.6, 4 d.f., *P* = 0.048).

### Seroprevalence Patterns by Age Class, Sex, and Year of Sampling

In city-wide sampling 2001–2005, marked big brown bats ranked from highest to lowest seroprevalence in the order adult females>volant juveniles>adult males (Table 2; overall *χ* ^2^ = 29.3, 2 d.f., *P*<0.0001). This pattern generally held within years. Females had higher seroprevalence than adult males (*χ* ^2^ = 10.0, 2 d.f., *P* = 0.007) or volant juveniles (*χ* ^2^ = 31.9, 2 d.f., *P*<0.0001). Seroprevalence did not differ between juveniles and adult males (*χ* ^2^ = 0.08, 2 d.f., *P* = 0.961). Seroprevalence in adult females varied among years (Table 2; *χ* ^2^ = 94.6, 4 d.f., *P*<0.0001). Adult females sampled in year 2003 had higher seroprevalence than in any other year except 2002; adult females sampled in 2004 had lower seroprevalence than in 2002 (*χ* ^2^ = 53.1, 4 d.f., *P*<0.0001) and 2003 (*χ* ^2^ = 100.1, 4 d.f., *P*<0.0001; Table 2). Year-to-year differences in overall seroprevalence of adult males (with smaller sample sizes) also were evident (*χ* ^2^ = 15.3, 4 d.f., *P* = 0.004; Table 2), largely due to contrasts of seroprevalence of adult males in 2003 with seroprevalence in 2002 (*χ* ^2^ = 10.8, 4 d.f., *P* = 0.029) and 2004 (*χ* ^2^ = 10.8, 4 d.f., *P* = 0.029). Volant juveniles had higher seroprevalence during 2003 than during all years except 2005, with juveniles in no other years differing significantly among each other (Table 2; overall *χ* ^2^ = 53.0, 4 d.f., *P*<0.001).

**Table 2:** Cross-sectional summaries of seroprevalence of RVNA by age class and sex in individual big brown bats (*Eptesicus fuscus*) by year, Fort Collins, Colorado, 2001–2005, all roosts combined.

Sex/Age	Statistic	2001	2002	2003	2004	2005	All Years
AF	%	12.3^a^	22.0^ b^	26.4^b^	6.0^a^	11.9^a^	17.9^x^
	*CI*	8.9–16.6	18.6–25.9	23.3–29.8	3.9–9.0	9.0–15.6	16.4–19.5
	*N*	310	518	734	368	402	2,332
AM	%	3.6^a,b^	0.0^a^	25.8^b^	0.0^a^	10.7^ab^	9.4^y^
	*CI*	0.1–20.2	0–18.5	12.5–44.9	0–20.9	2.8–29.4	5.2–16.1
	*N*	28	22	31	19	28	128
VJ	%	3.2^a^	6.3^a^	21.1^b^	2.6^a^	10.0^ab^	10.2^ y^
	*CI*	1.4–6.7	3.4–11.3	16.4–26.7	0.4–9.8	0.5–45.9	8.1–12.6
	*N*	220	174	256	78	10	738
Roosts	N	11	19	23	19	15	34

Proportions with superscripts in common for years across rows within age and sex categories, and among sex and age categories all years combined (right hand column) are not significantly different (*P*>0.05). Abbreviations: AF = adult females, AM = adult males, VJ = volant juveniles (sexes combined), *CI = *95% confidence interval for proportion, *N* = sample size, % = per cent seropositive.

### Seroprevalence in Adult Females by Roost

At the five roosts sampled in all five years total seroprevalence in adult females across years varied from 6.2 to 26.7% (Table 3; overall *χ* ^2^ = 31.5, 4 d.f., *P*<0.0001). Seroprevalence at one roost (#29) differed significantly from the other four roosts (P≤0.017), and seroprevalence values in bats from two other roosts (#58 and #60) were significantly different from each other (P = 0.03). No other pair-wise comparisons of seroprevalence at roosts showed significant differences. This pattern appeared to be held qualitatively for seroprevalence at roosts within single years, but with widely overlapping *CIs* for seroprevalence. The pattern among years seen in the city-wide sampling (Table 2) also appeared to hold for this subset of five roosts (Table 3; overall *χ* ^2^ = 49.9, 4 d.f., *P*<0.0001): seroprevalence was significantly higher in 2003 than during all other years (P<0.003) except 2002 (P = 0.408); adult females sampled in 2004 had lower seroprevalence than in 2002 (*χ* ^2^ = 22.6, 4 d.f., *P* = 0.0002) and 2003 (*χ* ^2^ = 53.1, 4 d.f., *P*<0.0001; Table 3). No other pair-wise comparisons of years showed significant differences.

**Table 3:** Seroprevalence of rabies virus neutralizing antibodies in individual adult female big brown bats (*Eptesicus fuscus*) within roosting colonies by year at five roosts, Fort Collins, Colorado, 2001–2005.

Roost ID #	Statistic	2001	2002	2003	2004	2005	All Years
#58 (219)	%	10.4	19.8	31.2	7.2	10.9	16.9^ x^
	*CI*	4.9–20.0	12.0–30.4	23.0–40.8	3.0–15.7	5.6–19.5	13.6–20.7
	*N*	77	81	112	83	92	445
#60 (203)	%	28.3	32.3	34.9	7.0	18.9	26.7^z^
	*CI*	17.2–42.6	21.6–45.2	25.0–46.3	1.8–20.1	8.6–35.7	21.7–32.3
	*N*	53	65	83	43	37	281
#29 (23)	%	6.4	7.7	10.4	0.0	0.0	6.2^y^
	*CI*	1.1–22.8	1.3–26.6	3.9–23.4	0.0–17.2	0.0–22.9	3.0–11.7
	*N*	31	26	48	24	17	146
#44 (77)	%	16.7	20.5	34.3	0.0	22.2	20.8^x z^
	*CI*	8.0–30.8	10.3–35.7	19.7–52.3	0.0–26.8	7.4–48.1	14.9–28.0
	*N*	48	44	35	14	18	159
#51 (126)	%	13.3	23.1	32.7	16.0	14.3	22.4^xz^
	*CI*	2.3–41.6	9.8–44.1	20.4–47.6	5.2–36.9	4.7–33.6	16.0–30.3
	*N*	15	26	49	25	28	143
All Roosts	%	15.6^ac^	22.3^ab^	29.7^b^	6.9^c^	13.0^ac^	19.1
	*CI*	11.3–21.2	17.3–28.2	24.8–35.0	3.9–11.7	8.8–18.8	16.9–21.5
	*N*	224	242	327	189	192	1,174

Proportions with superscripts in common for roosts within the right-hand column or for years across the bottom row are not significantly different (P>0.05). *CI = *95% confidence interval for proportion, % = per cent seropositive, *N* = sample size. Maximum count of adults as a measure of relative colony size (see Methods) is given in parentheses following roost identifiers. Seroprevalence by roost across all years is given in the right hand column, and by year across all roosts in the bottom row, with seroprevalence across all roosts and years provided in the lower right corner.

Variability in seroprevalence at 17 other roosts sampled for fewer than five summers ranged from 0.0 to 41.7% during individual years (Table S1). Efforts at roosts varied among years, making comparisons across years and roosts less straightforward to interpret. However, the presence of RVNA was widespread. RVNA were detectable in each year of study, and in each roost across the years sampled (Table S1). Within individual years and roosts, seropositive bats were detected in 36 of 49 summer samplings (Table S1). Within years, *CIs* for seroprevalence at individual roosts all were widely overlapping. Across years sampled in common, pooled seroprevalence at individual roosts also showed widely overlapping *CIs* (e.g., *cf*. roosts in 2003–2005; Table S1).

### Factors Influencing Serological Status of Individuals

**Figure 2:**
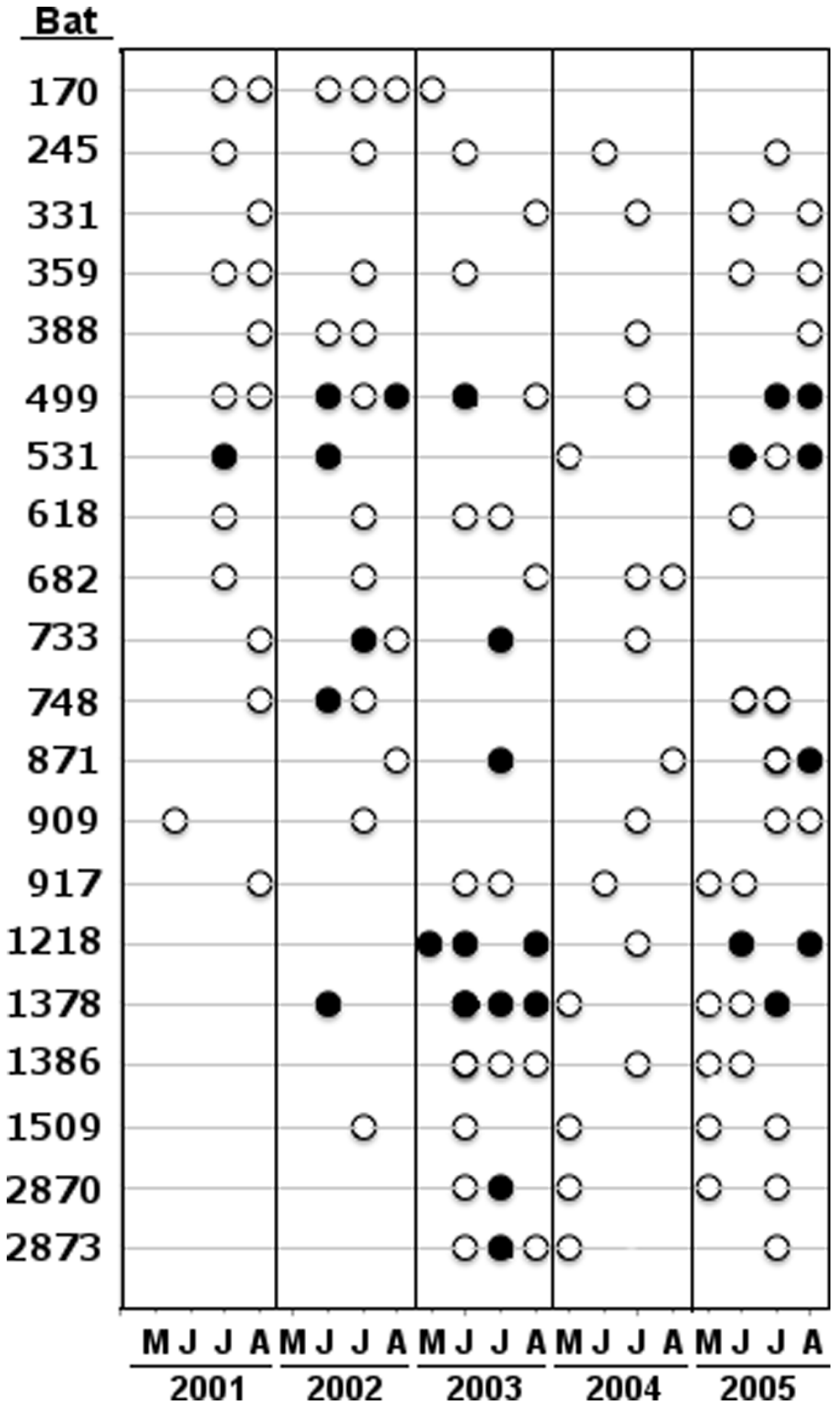
Examples of temporal variability in RVNA serological status of 20 wild big brown bats (*Eptesicus fuscus*) sampled 5 or more times, Fort Collins, Colorado 2001–2005. Open circles denote seronegative, closed circles denote seropositive at date shown at the bottom of the figure (vertical lines separate years). M-A are months of May through August each year. Individual bat identification number is given at the left-hand column.

**Figure 3:**
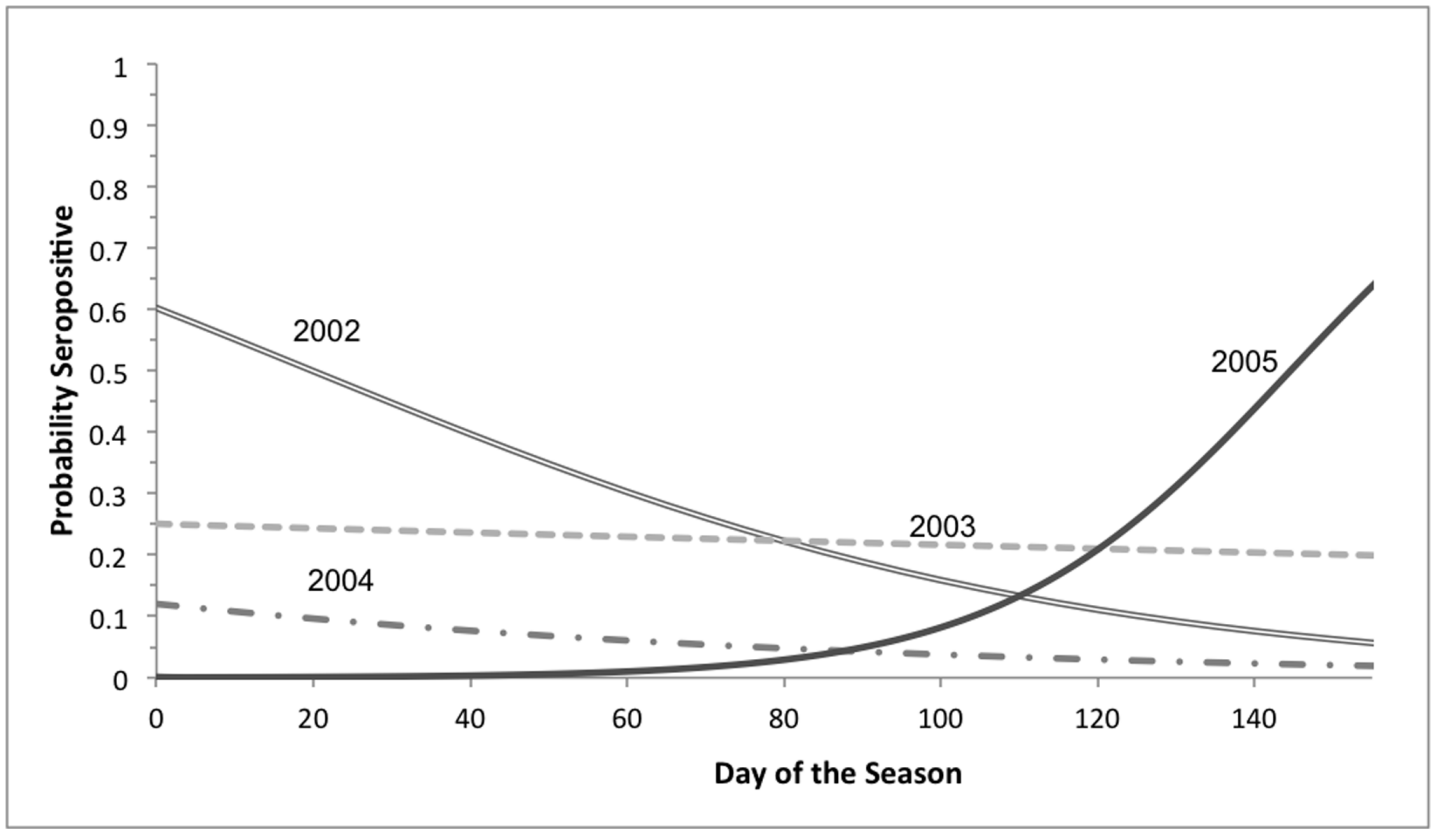
Relationship between day of the season (horizontal axis) and probability of an adult female big brown bat (*Eptesicus fuscus*) being seropositive for RVNA on first sampling in three different years of study. The reference year is 2003.

The presence of RVNA in individual bats showed temporal variability, sometimes after short intervals between samples (Figure 2). In our logistic regression analysis of the factors influencing the serological status of the bat when it was first sampled, the highest ranking model in our candidate set had an intercept (*β*_0_), main effects for year (*β_yr_*) and day of season (*β_dos_*), and a year x day of season interaction term (β_yr*dos_) (*w_ = _*0.670, *k* = 8, *n* = 1,343 observations). No other models were competitive (i.e., ΔAIC*_c_* >8 for all other models). Diagnostic plots suggested that the interaction was complex and varied with year (Figure 3). In comparison with 2003, which was the reference year in this analysis (*i.e*., *β* [95% CI]; *β*_0_ = −1.097 [−1.796 to −0.391], *β_yr_* = 0, *β_dos_* = −0.002 [−0.011 to 0.006], and β_yr*dos_ = 0), the likelihood of a bat being seropositive in 2005 began lower and increased as the season progressed (*β*_0_ and *β_dos_* are unchanged, *β_yr_* = −6.741 [−12.296 to −2.984], β_yr*dos_ = 0.056 [0.019 to 0.105]). In 2002 the likelihood of a bat being seropositive began higher than in 2003 and continued to decrease as the season progressed (*β*_0_ and *β_dos_* are unchanged, *β_yr_* = 1.510 [0.106 to 2.936], β_yr*dos_ = −0.019 [−0.036 to −0.002]), whereas in 2004 the likelihood of a bat being seropositive began lower than in 2003 but also decreased as the season progressed (Figure 3; *β*_0_ and *β_dos_* are unchanged, *β_yr_* = −0.893 [−3.931 to 1.866], β_yr*dos_ = −0.011 [−0.045 to 0.020]). Although overall the PDSI for the April-September period in 2005 documented drought conditions, the PDSI in months of June and July 2005 was normal, whereas in both 2002 and 2004 drought was persistent throughout the summer months (Figure 1).

**Figure 4:**
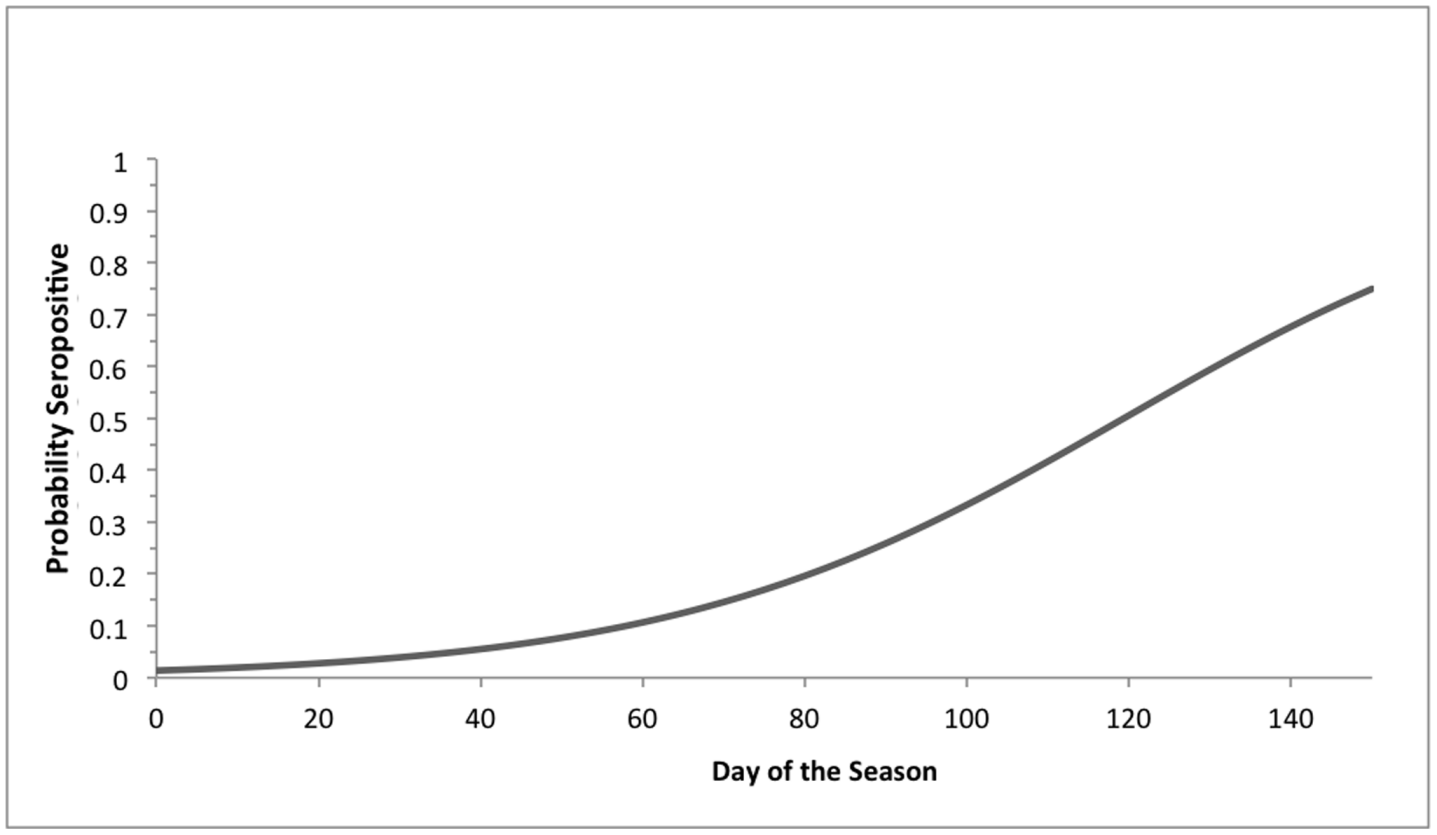
Probability of an adult female big brown bat (*Eptesicus fuscus*) transitioning from seronegative to seropositive for RVNA with the advancing season of bat activity (early April to September), conditional that the bat was seronegative on the previous sampling (see Methods).

In our logistic regression analysis of the likelihood of an adult female transitioning to seronegative from a previously seropositive state, the highest ranking model in our candidate set depended only on year (*w* = 0.906, *k* = 4, *n = *169 observations). Parameter estimates [95% CI] indicated that the likelihood of this transition was higher in 2004 (*β* = 1.280 [0.464 to 2.143]) and 2005 (*β* = 1.727 [0.899 to 2.624]) relative to 2003, but not in 2002 (*β* = −0.713 [−2.303 to 0.633]). In our logistic regression analysis of the likelihood of an adult female transitioning from seronegative to seropositive, the highest ranking model in our candidate set depended only on day of the season (*w* = 0.410, *k* = 2, *n* = 52 observations). This likelihood increased as the season progressed (Figure 4; *β = *0.036 [0.011 to 0.065]).

### Lack of Rabies Viral RNA in Bats

We tested 467 samples of oropharyngeal secretions from 363 individual bats for the presence of RV RNA. Only one sample was positive, from a bat that was seronegative on the day of sampling. This bat was never captured again, and was never detected by an automated tag reader in place at its roost each summer for the next four years. RV genome-specific N gene RNA was not detected in any of 293 oropharyngeal secretions from a subsample of 261 individuals that were all seropositive on the dates of testing (32 individuals were sampled on >1 date).

The remaining negative samples were from bats that were seronegative at the time of sampling (106 oropharyngeal secretion samples from 98 individual bats) or did not have serological status determined on the date of sampling (67 secretion samples from 66 individuals). Among the bats in these latter two groups were individuals that had negative oropharyngeal samples but were seropositive for RVNA earlier during the same summer (34.1±19, range 13–73 days prior to oropharyngeal sampling, *n* = 21 intervals from 19 individuals) or during the summer of the previous year (8 intervals from 6 individuals).

Samples of total RNA from salivary glands and brains of 13 adult female bats that were seropositive during summer 2001 were assayed after recapture during 2002. None of the brain or salivary gland preparations from these bats was positive for RV genome-specific N gene RNA. Eleven of the 13 were also sampled for serology when recaptured in 2002, with nine seropositive on recapture (1 seronegative bat recaptured in 2002 was a volant juvenile when sampled in 2001). Similarly, an additional bat was seropositive in June 2002 and again when recaptured 34 days later, but had no detectable RV genome-specific N gene RNA in brains or salivary glands at the date of final recapture. In addition to these samples, two sick bats found near roosts were diagnosed as rabid based on DFA analysis of brain tissue: one was seronegative and one was seropositive on the day they were euthanized.

### Survival of Seropositive Bats

The annual survival estimate of adult female bats during 2001–2005 that were seropositive during 2001–2003 (*φ* = 0.85 [95% *CI* 0.80 to 0.89], *n* = 360 bats) showed no significant difference (*P = *0.783, *χ* ^2^ = 4.0, 7 d.f.) with that of bats only known to be seronegative during this period (*φ* = 0.83 [95% *CI* 0.81 to 0.86], *n* = 106 bats).

Ten of 15 bats at regularly monitored roosts that were seropositive in 2001 were known to be alive in 2005, and 39 of 55 bats seropositive in 2002 were known to be alive in 2005. Bats captured haphazardly in nets during limited efforts for the unrelated study in 2007 and 2008 included 10 individuals that were known to be seropositive as adults 5 years earlier, 4 individuals seropositive as adults 6 years earlier, and one bat seropositive as an adult 7 years earlier.

## Discussion

### Seroprevalence in Volant Juveniles and Known-Age Adult Females

It is likely that many of the seropositive volant juveniles exhibited passive immunity from maternally derived antibodies. This also was reported for juvenile Mexican free-tailed bats [2]. In big brown bats at Fort Collins, one-year-old females had 14% seroprevalence, whereas RVNA were not detected in one-year-old males. Unlike breeding adult females, adult male big brown bats typically do not roost in clustering groups [66] that might foster greater exposure to RV due to higher densities of bats. Cumulative evidence for RVNA in serum ranged from 21 to 48 percent in adult females that were 2 years old or older, indicating that exposure of big brown bats to RV is common. The proportion seropositive was significantly lower in one-year olds compared to four-year olds; a tendency for increased seroprevalence with age is consistent with the finding that in this population one-third of one-year-old females do not breed, whereas nearly all older females breed [26]. Although occupying the same maternity roosts as breeders, non-breeding one-year olds may not exhibit a strong tendency to engage in clustering with its presumed increased likelihood of exposure to RV.

### Seroprevalence Patterns by Sex, Age Class, and Year of Sampling

Adult females consistently showed higher seroprevalence than adult males. This pattern generally held within years, and is concordant with the greater degree of coloniality and likely exposure to RV in females. However, differences in seroprevalence between males and females have not been observed consistently in the few other species of North American insectivorous bats that have been sampled for RVNA. Differences in seroprevalence between sexes were not apparent in adult Mexican free-tailed bats at colonies in Texas or at Carlsbad Caverns, New Mexico [2], [29]. Adult females may tend to have higher seroprevalence than adult males in hoary bats (*Lasiurus cinereus*), but not in silver-haired bats (*Lasionycteris noctivagans*), little brown bats (*Myotis lucifugus*), or long-legged myotis (*M. volans*) [50].

Seroprevalence in adult female big brown bats varied among years in city-wide sampling. Most notably, adult females sampled in 2003 had highest seroprevalence (but not significantly different from 2002), and those sampled in 2004 had the lowest seroprevalence (but not significantly different from 2001 and 2005). This pattern also held at the five roosts sampled each year. Volant juveniles also had higher seroprevalence during 2003 (but similar to 2005) in city-wide sampling, presumably because a greater proportion expressed maternally derived antibodies, paralleling higher adult female seroprevalence. Year-to-year differences in overall seroprevalence within adult males (with much smaller sample sizes) also followed this pattern in part, with higher seroprevalence in 2003 (but not significantly different than 2005) and lowest in 2004 (but not significantly lower than during years other than 2003).

The year-to-year pattern in seroprevalence roughly followed the annual variation in drought severity affecting the Fort Collins region, suggesting that lower production of RVNA in times of environmental stress may contribute to variability in RVNA seroprevalence. The year 2003 had reduced drought conditions during the April-September period of bat activity compared to other years, although in 2005 June and July were more favorable (Figure 1). Drought occurred to a greater degree in all other years, with 2002 the greatest and 2004 the next greatest in drought severity. Although seroprevalence was higher in 2002 than 2004, we speculate that the lower seroprevalence in 2004 compared to 2002 may have been exacerbated by other factors with potential stress on the big brown bat population. In 2004, areas used for foraging by big brown bats [25] were subject to much more intensive mosquito control targeting vectors in a novel West Nile virus epizootic that had recently reached Colorado [67]. This included use of pyrethroid and organophosphate insecticides as well as mosquito larvicides [68], [69]. Effects of modern insecticides on bats or their specific food supplies are poorly known [70], but insect control may have been an additional environmental stress in 2004 that was not present in 2002. Dietary analysis shows that big brown bats consumed prey from different orders of insects in 2004 than in other years, including a large proportion of small midges instead of more typical dietary items such as large beetles (E. Valdez, U.S. Geological Survey, unpublished observations). Also in 2004, the month of July was notably cooler than in other years (Table 4), with nursing bats and suckling young found in torpor during sampling at roosts (unpublished observations). We are uncertain why seroprevalence in the drought year of 2002 was not significantly lower than seroprevalence during 2003, although temperatures were considerably warmer in June 2002 than in June of other years (Table 4) and warmer conditions can positively affect expression of antibodies in bats [57].

**Table 4:** Summary statistics for ambient temperatures (^o^C) during June and July 2001–2005, Fort Collins, Colorado: means of daily maximum temperatures (*X¯_max_* ) and their lower and upper 95% confidence limits (*CL_max_*), the range of daily maxima (range_max),_ and the number of days each month with temperatures exceeding 32°C (*N* days >32°C).

	2001	2002	2003	2004	2005
JUNE *X¯_max_*	27.6	29.5	23.1	23.3	25.8
JUNE *CL*_max_	25.6, 29.6	27.9, 31.2	21.7, 24.6	21.3, 25.4	23.9, 27.8
JUNE range*_max_*	16.4–35.4	17.2–34.9	13.4–29.7	10.2–35.9	14.3–32.4
JUNE *N* days >32°C	10	15	0	3	5
JULY *X¯_max_*	30.7	32.2	32.7	27.9	32.0
JULY *CL*_max_	29.6, 31.7	31.2, 33.2	31.6, 33.8	26.0, 29.7	30.6, 33.4
JULY range*_max_*	26.0–35.6	25.8–37.4	26.7–37.2	14.6–35.5	19.1–38.5
JULY *N* days >32°C	15	21	22	11	22

### Seroprevalence in Adult Females by Roost

Results of sampling at 17 roosts for variable numbers of years support previous conclusions [14], [50] that the presence of antibodies and exposure to RV are widespread among big brown bat colonies in the region. Differences in seroprevalence among most roosts were not readily apparent. However, at the five roosts sampled in each of the five years of study, seroprevalence was consistently and significantly lower at one roost (#29, Table 3). We are uncertain why this was so. However, bats at this roost also had lower adult and first-year survival [26], [27]. They also occurred in a smaller colony than at the other four roosts, perhaps reducing chances of exposure to RV. The annual variation in seroprevalence at the five roosting colonies also followed the year-to-year patterns seen in the city-wide sampling: highest seroprevalence in 2003 and lowest in 2004.

Ranges of seroprevalence at roosts of big brown bats in Fort Collins were similar to the reported ranges of seroprevalence for this species from colonies in New York in 1973–1976, but with higher overall seroprevalence (0.0 to 40%, overall 9.6% in New York; [3]), and similar to seroprevalence at other roosts in Colorado during 2003 (5.6 to 54.6%; [50]). Seroprevalence in big brown bats was higher than that reported for little brown bats (*Myotis lucifugus*) at colonies in New York (0.0 to 6.9%, overall 2.4%; [3]) and in little brown bats captured at foraging areas in Colorado (0.0 to 8.3%, overall 2.9%; [50]). Seroprevalence can be as high as 80% in adult female Mexican free-tailed bats [5], [29], [71]. Seroprevalence of RVNA in adult females of the largely solitary hoary bat was 31.8%, and seroprevalence in silver-haired bats was 6.8% in Colorado and New Mexico [50].

### Factors Influencing Serological Status of Individuals

Relatively high fidelity of big brown bats to roosts within core areas [25], [48] facilitated repeated serological sampling of individual bats. The degree of waxing and waning of detectable RVNA observed in repeatedly recaptured individuals was not anticipated. It suggests that many adult big brown bats may be immunologically primed against RV due to past exposures, are not actively producing antibodies at the time of sampling, but may exhibit anamnestic responses on subsequent exposures. If this is true, then cross-sectional surveys underestimate the amount of exposure to RV that is encountered by bats. An absence of RVNA production also may be due to other demands on the immune system, or environmental and physiological factors as suggested for other wildlife [30], [31]. Although re-exposure may be a trigger to renewed RVNA production in big brown bats, we were not able to document or measure such events in the field. However, the likely capability of many seemingly seronegative bats to exhibit anamnestic responses [72] on subsequent exposures to RV would provide some immunity and result in a lack of observations of epizootic increases in rabies deaths among big brown bats during this and other studies [14], [25], [73] (see also “Comparisons with Captive Exposure Experiments” below).

The environmental and physiological factors in our models for serological status on first sampling did not implicate female reproductive condition, recent biting of wings, short term ambient temperatures prior to sampling, and days elapsed since prior sampling as factors associated with serological status. However, an interaction of year and day of the season at the time of sampling influenced the likelihood of an adult female being seropositive on first capture. When compared to 2003 (near normal temperature and moisture conditions and highest cross-sectional seroprevalence), bats were more likely to be seropositive during 2005, the drought year that had near-normal summer temperature and moisture in June and July, than during 2002 and 2004, drought years that remained persistently dry during summer.

Year and day of the season were also implicated in affecting the probabilities of individual bats changing serological status, but in different ways. Adult female big brown bats were more likely to have shown a shift from seropositive to seronegative during 2004 and 2005 in comparison with 2003. Day of the season was implicated as the only factor associated with bats making a transition from a prior seronegative to a seropositive state. This suggests that as the active season progresses in any year, the cumulative likelihood of a bat being exposed to RV and expressing RVNA also increases. This is consistent with models of the dynamics of RV infection in big brown bats from throughout the United States, which show seasonally increasing incidence of rabies cyclically each year [35].

### Comparisons with Captive Exposure Experiments

Results from experimental RV infections in captive big brown bats are pertinent to our findings on waxing and waning of RVNAs. Seven wild big brown bats that were seropositive when captured maintained titers during 5 months in captivity and had no RV antigen in brains when euthanized [17]. Three other bats had no RVNA in one of six monthly samplings each, indicating a waxing and waning of antibody production [17]. Two juveniles with positive titers shortly after being brought into captivity during late summer were seronegative at five monthly bleedings thereafter, suggesting waned passive immunity [17]. Substantial antibody titers could be induced in big brown bats through non-lethal exposures to RV or inoculation with RV vaccine, leading to the hypotheses that antibodies were indicative of some degree of immunity, and that bats experience such exposures in the wild [17] (perhaps by receiving small loads of virus transmitted through bites in distal regions, or in transfer of virus in saliva of infected bats in prodromal stages of the disease while scratching and grooming in clusters with non-infected bats). Jackson et al. [18] found that 17 of 20 big brown bats experimentally inoculated with high doses of RV showed seroconversion. Four bats that survived experimental inoculation maintained titers for >three months, but titers then declined to non-detectable levels at 4.6 months post-exposure. Jackson et al. [18] also suggested that the immune response of big brown bats can prevent a productive RV infection. These findings were followed by experimental inoculation of big brown bats at several dosage levels, then re-inoculation of survivors in secondary and tertiary experiments at 175 and 305 days after the initial exposure [19]. Regardless of dosage, at each experimental stage bats that seroconverted after inoculation had a higher probability of surviving than those that did not, and bats that succumbed but showed RVNA did not seroconvert until 1–6 days before death [19]. Titers in RVNA-positive bats peaked from 31–42 days post-exposure after the first inoculation, but after 175 days 23 of 26 (88%) bats were seronegative; titers remained detectable 6–12 months after secondary inoculation [19]. On the third experimental inoculation (349 days after the first and 174 days after the second), survivors (6 of 10) had antibody titers prior to injection, and showed significantly lower mortality, leading to the conclusion that long-term repeated inoculation of big brown bats with RV may indeed confer significant immunological memory and reduced susceptibility to RV infection [19]. In another experiment, captive big brown bats from our study population were inoculated with either of two big brown bat RV variants circulating locally [22]. Bats that were seropositive when brought into captivity all survived an intramuscular challenge with 10^3^ TCID 50 dose of one variant without developing clinical rabies, and subsequently showed increased circulating RVNA; most bats that were seronegative on inoculation with this variant developed fatal rabies [22]. Big brown bats inoculated with the second RV variant all survived without developing rabies, and most seroconverted but with RVNA becoming undetectable after 90 days post-inoculation [22]. Bats that were naturally seropositive at the time of capture did not develop rabies when inoculated with this second variant [22].

Our studies of RVNA in wild big brown bats concur with the above experimental findings in several respects. In the experimental studies captive bats also maintained antibodies for variable periods, with many seropositive for about 5 months, but RVNA were more persistent with repeated exposure [17]–[19]. We found that some wild individuals consistently showed titers over time whereas others showed variability in RVNA production. Such individual variability may be due to frequency and nature of re-exposures to RV in the wild, as well as to individual variability in general health and susceptibility to environmental stressors. Constantine [71] suggested that some bats may be less susceptible to RV infection and those that develop the disease may be immunocompromised. It is of note that seronegative bats at our study area also had greater infestation by some ectoparasites than seropositive bats, perhaps correlated with greater stress [74]. Experiments demonstrated that wild seropositive bats can have immunity to experimental infection, and that experimental inoculation with a variant of RV found in our study population can lead to circulating RVNA without the disease [22], adding another dimension to the complexity of host-virus dynamics in this system. Bats held in captivity with constant environmental conditions and ready access to food and water in experimental studies are less subject to variable environmental influences, and may show less temporal variability and perhaps longer periods of RVNA production than wild big brown bats. Survival rates and absence of evidence for RV or RV RNA in seropositive wild big brown bats (first demonstrated by Trimarchi and Debbie [3]), and a preponderance of seronegative individuals in most experimentally infected rabid bats (other than cases shortly before death) suggest that in most cases the presence of RVNA in serum should be interpreted primarily as evidence of prior exposure rather than an ongoing RV infection. However, much variability can be expected in expression of RVNA in relation to infection status in big brown bats, as is observed in other mammals [75].

We hypothesized that the phase of reproduction might be an important stress factor influencing seroprevalence. We also anticipated that recent temperature conditions might also influence the presence of RVNA because of implications for daily torpor and its influence on metabolic rates that can in turn influence the immune system of mammals [57]–[60], [76]. Instead we found no support for these predictions, and suggest that variability in RVNA production may be more influenced by year-to-year differences in environmental conditions, especially drought. Prolonged drought may mimic chronic stress, which can impair IgG production and other aspects of immune function in laboratory mice [77], [78]. Chronic stress also can elevate baseline cortisol in mammals, and in other species of bats baseline cortisol increases during seasonal food scarcity and periods of poor body condition [79], [80]. Although cortisol effects on immunity can be complex, chronic elevation of cortisol can be immunosuppressive [81], [82]; restricted energy availability also has negative effects on immune function [83]. We are unaware of any studies of the impact of drought on stress and immune responses of big brown bats. However, some data exist on such responses in other wildlife due to drought, and droughts are associated with negative demographic effects in bats that imply a chronic health effect. Drought had negative impacts on cell-mediated immunity, clutch size, and body mass in passerine birds in New Mexico [84]. Elevated cortisol and impaired innate immunity was documented in wild wallaroos (*Macropus robustus*) during a major drought in Western Australia [85]. Reduced fecundity during a drought year in comparison with a normal year was reported for 3 species of insectivorous bats in southwestern Colorado [40], and female white-striped bats (*Tadarida australis*) ceased reproduction during drought in Australia [86]. Drought years are associated with lower survival of little brown bats in New Hampshire and lower survival and reduced population growth rates in Yuma myotis (*Myotis yumanensis*) in California [39], [87]. Drought may exert these demographic effects on bats through decreased insect abundance and resultant energetic and metabolic constraints [39], [40]; mounting of immune responses also can be metabolically costly [83], [88]. Given this background, we hypothesize that chronic prey shortage and energetic restriction may contribute to suppressed antibody production in big brown bats.

Our hypothesis that drought may be one of the environmental stressors of importance to RVNA production in big brown bats has negative implications in the context of global climate change. Predictions of global climate change models forecast greater drought conditions throughout much of Western North America [89]–[91]. Drought has been associated with negative impacts on demographic traits of insectivorous bats as noted above. Our findings suggest the hypothesis that drought has additional negative implications for health of insectivorous bat populations. Alternatively, big brown bats at our study area by coincidence may have been exposed to RV to a greater extent during years when greater seroprevalence was observed. However, passive public health surveillance data and other observations from the study area did not indicate any major changes in the number or proportion of bats with rabies during any year of our study [25].

## Supporting Information

Table S1 **Seroprevalence of rabies virus neutralizing antibodies in individual adult female big brown bats (*Eptesicus fuscus*) from 17 roosts sampled for 1–4 summers, Fort Collins, Colorado, 2001–2005.**
(DOCX)
