# Supplementary material for: Variability in Seroprevalence of Rabies Virus Neutralizing Antibodies and Associated Factors in a Colorado Population of Big Brown Bats (Eptesicus fuscus)
Source: PLoS One. 2014 Jan 22;9(1):e86261. doi: 10.1371/journal.pone.0086261 (PMC3899234; doi:10.1371/journal.pone.0086261)
Supplement: Table S1 — Seroprevalence of rabies virus neutralizing antibodies in individual adult female big brown bats (Eptesicus fuscus) from 17 roosts sampled for 1–4 summers, Fort Collins, Colorado, 2001–2005. (DOCX) [file pone.0086261.s001.docx]

**Table S1**. **Seroprevalence of rabies virus neutralizing antibodies in individual adult female big brown bats (*Eptesicus fuscus*) from 17 roosts sampled for 1-4 summers, Fort Collins, Colorado, 2001-2005.**

| **Roost ID #** | **Statistic** | **2001** | **2002** | **2003** | **2004** | **2005** | **All Years** |
| --- | --- | --- | --- | --- | --- | --- | --- |
| #133 (156) | % | 2.0 | 28.8 | 33.3 | 0.0 | ND | 18.1 |
|  | *CI* | 0.1-11.8 | 19.1-40.7 | 6.0-75.9 | 0.0-69.0 |  | 12.1-25.9 |
|  | *N* | 51 | 73 | 6 | 3 | ND | 133 |
| #4 (62) | % | 0.0 | 22.2 | 25.7 | ND | ND | 22.4 |
|  | *CI* | 0.0-53.7 | 7.4-48.1 | 13.1-43.6 | ND | ND | 2.8-17.2 |
|  | *N* | 5 | 18 | 35 | ND | ND | 58 |
| #131 (31) | % | 0.0 | 4.0 | 14.3 | ND | ND | 7.45 |
|  | *CI* | 0.0-26.8 | 0.2-22.3 | 4.7-33.6 | ND | ND | 2.8-17.2 |
|  | *N* | 14 | 25 | 28 | ND | ND | 67 |
| #57 (54) | % | ND | 13.8 | 24.4 | 0.0 | 6.2 | 15.5 |
|  | *CI* | ND | 4.5-32.6 | 12.9-40.6 | 0.0-32.1 | 0.3-32.3 | 9.2-24.5 |
|  | *N* | ND | 29 | 41 | 11 | 16 | 97 |
| #56 (60) | % | ND | 14.3 | 27.0 | 4.4 | 0.0 | 16.4 |
|  | *CI* | ND | 2.5-43.8 | 16.9-39.9 | 0.2 – 24.0 | 0.0-18.5 | 10.5-24.4 |
|  | *N* | ND | 14 | 63 | 23 | 22 | 122 |
| #139 (30) | % | ND | 0.0 | 25.0 | 0.0 | 21.4 | 15.9 |
|  | *CI* | ND | 0.0 – 43.9 | 8.3 – 52.6 | 0.0 – 43.9 | 5.7-51.2 | 7.2 -30.7 |
|  | *N* | ND | 7 | 16 | 7 | 14 | 44 |
| #69 (25) | % | ND | 40.0 | 41.2 | 0.0 | 22.2 | 30.0 |
|  | *CI* | ND | 13.7-72.6 | 19.4-66.6 | 0.0-53.7 | 7.4-48.1 | 18.3-44.8 |
|  | *N* | ND | 10 | 17 | 5 | 18 | 50 |
| #4 (34) | % | ND | 0.0 | 5.0 | 0.0 | ND | 2.8 |
|  | *CI* | ND | 0.0-28.3 | 0.2 -26.9 | 0.0-69.0 | ND | 0.1-16.2 |
|  | *N* | ND | 13 | 20 | 3 | ND | 36 |
| #97 (54) | % | ND | 41.7 | 26.3 | ND | ND | 32.3 |
|  | *CI* | ND | 16.5-71.4 | 10.1-51.4 | ND | ND | 17.3-51.5 |
|  | *N* | ND | 12 | 19 | ND | ND | 31 |
| #118 (105) | % | ND | 31.1 | ND | ND | ND | 31.1 |
|  | *CI* | ND | 20.3-44.4 | ND | ND | ND | 20.3-44.4 |
|  | *N* | ND | 61 | ND | ND | ND | 61 |
| #138 (46) | % | ND | ND | 16.7 | 0.0 | 3.4 | 8.1 |
|  | *CI* | ND | ND | 7.0-33.5 | 0.0-19.2 | 0.2-19.6 | 3.6 -16.6 |
|  | *N* | ND | ND | 36 | 21 | 29 | 86 |
| #126 (45) | % | ND | ND | 33.3 | 0.0 | 0.0 | 15.5 |
|  | *CI* | ND | ND | 17.2 – 54.0 | 0.0-26.8 | 0.0-22.9 | 7.8-27.9 |
|  | *N* | ND | ND | 27 | 14 | 17 | 58 |
| #182 (ND) | % | ND | ND | 19.2 | 9.5 | 31.6 | 19.7 |
|  | *CI* | ND | ND | 7.3-40.0 | 1.7-31.8 | 13.6-56.5 | 11.3-31.7 |
|  | *N* | ND | ND | 26 | 21 | 19 | 66 |
| #28 (74) | % | ND | ND | 20.4 | 7.7 | ND | 17.7 |
|  | *CI* | ND | ND | 10.7-34.8 | 0.4 – 37.9 | ND | 9.6-29.9 |
|  | *N* | ND | ND | 49 | 13 | ND | 62 |
| #49 (35) | % | ND | ND | 20.8 | 22.2 | ND | 21.2 |
|  | *CI* | ND | ND | 7.9-42.7 | 4.0 – 59.8 | ND | 9.6-39.4 |
|  | *N* | ND | ND | 24 | 9 | ND | 33 |
| #141 (179) | % | ND | ND | ND | 5.9 | 15.2 | 12.7 |
|  | *CI* | ND | ND | ND | 0.3-30.8 | 6.8-29.5 | 6.0-24.1 |
|  | *N* | ND | ND | ND | 17 | 46 | 63 |
| #140 (82) | % | ND | ND | ND | 3.9 | 3.4 | 3.6 |
|  | *CI* | ND | ND | ND | 0.2-21.6 | 0.2-19.6 | 0.6-13.6 |
|  | *N* | ND | ND | ND | 26 | 29 | 55 |
| All Roosts | % | 1.4 | 22.9 | 23.0 | 4.6 | 11.0 | 16.7 |
|  | *CI* | 0.1-9.0 | 18.0-28.6 | 19.1-27.5 | 2.2-9.2 | 7.2-16.2 | 14.5-19.0 |
|  | *N* | 70 | 262 | 408 | 173 | 210 | 1,123 |

Abbreviations: *CI* = 95% confidence interval for proportion, *N* = number of individuals sampled, ND = not determined, % = per cent seropositive. Maximum counts of adults as a measure of relative colony size (see Methods) is given in parentheses following roost identifiers. Seroprevalence by roost across all years is given in the right hand column, and by year across all roosts in the bottom row, with seroprevalence across all roosts and years provided in the lower right corner.
